# Empowering radiologists: a look at standardized reporting initiatives in India

**DOI:** 10.1186/s13244-024-01718-4

**Published:** 2024-07-25

**Authors:** Pranjal Rai, Abhishek Mahajan

**Affiliations:** 1 Department of Radiology, Tata Memorial Hospital, Homi Bhabha National Institute, Mumbai, India; 2 Department of Imaging, The Clatterbridge Cancer Centre NHS Foundation Trust, Liverpool, UK; 3 Faculty of Health and Life Sciences, University of Liverpool, Liverpool, UK

## Opinion on “ESR paper on structured reporting in radiology—update 2023”

In this article written by the European Society of Radiology (ESR), the focus of discussion has been the current trends with respect to structured reporting in radiology across the globe [1]. The section on the overview of current technical standards discusses the Integration of the Healthcare Enterprise Management of Radiology Reporting Templates (IHE MRRT) profile, which was integrated into the IHE’s radiological technical framework in early 2022 with the combined efforts of the Radiological Society of North America and the ESR. While this profile was not widely adopted, it did pave the way for more advanced interoperable formats, which may even allow for the incorporation of figures and links to imaging studies. The authors also agree that there is still a lack of agreement on technical standards.

The article also briefly explores the various initiatives undertaken by radiological societies across the globe to implement structured reporting formats in daily radiological practice on an institutional level. While the efforts taken by the authors are remarkable and the information conveyed is quite comprehensive, we believe that the subsection related to Turkey, Israel, and India did not accurately convey the trends of structured reporting that are being practiced, particularly in the Indian subcontinent. According to the authors, the radiologists in these countries were not aware of any national initiatives related to structured reporting, and neither were any incentives offered nor any mandates in place.

While the concern regarding a lack of national initiatives for structured reporting in some regions is valid, it’s important to acknowledge the efforts undertaken by professional organizations in others. In India, the Indian College of Radiology and Imaging (ICRI), the academic wing of the Indian Radiological and Imaging Association (IRIA), has taken a commendable step.

The ICRI’s subspecialty group for oncoimaging, under the lead authorship of Chaturvedi et al has developed a comprehensive set of standard imaging protocols and structured reporting templates/checklists encompassing various cancers throughout the body [2]. This first edition, released in August 2020, provides background information, imaging protocols, and checklist-style reporting templates for a wide range of oncological conditions.

The document has been extensively disseminated across major Indian institutions and adopted by radiologists nationwide. This facilitates seamless inter-institutional and inter-departmental communication of critical information not only for radiologists but also for surgical, medical, and radiation oncologists. Notably, the document was formulated in consultation with leading oncoimaging experts in India, ensuring its merit and credibility.

Furthermore, the Indian Journal of Medical and Pediatric Oncology, a bi-monthly peer-reviewed international journal published by the Indian Society of Medical and Pediatric Oncology—the apex body for medical and pediatric oncologists in India—has special issues, guest authored by Mahajan et al underscoring comprehensive imaging recommendations for diagnosing, staging, and managing various tumors throughout the body [3, 4]. These standardized imaging guidelines empower trained and in-training radiologists across India to integrate the protocols into their institutional workflows, ensuring the most accurate imaging and reporting for every patient.

The Comprehensive Textbook of Clinical Radiology, a 2023 initiative under the aegis of the IRIA, serves as a comprehensive reference for radiology residents and practicing radiologists [5]. Published internationally by Elsevier, this text provides a valuable resource for those seeking in-depth knowledge. Notably, subsection 2.15.14, authored by Mahajan et al, offers valuable details into imaging protocols and reporting formats in routine oncological practice.

While the article provides a valuable overview of international structured reporting initiatives, it may not fully capture the efforts undertaken in India. The ICRI comprehensive guidelines, while extensive, might not have been reflected in the author’s discussion. This could be partly due to limited awareness among radiologists surveyed for the article. Disseminating these guidelines across India’s diverse regions and languages remains a challenge.

Furthermore, leading radiologists from India’s premier cancer imaging institutes have significantly contributed to the development of standardized synoptic reporting formats for head and neck, lung, esophageal, and thyroid cancers [6–10]. These formats have facilitated radiologists at other institutions, to deliver accurate and concise information relevant to clinicians. This approach has streamlined communication and eliminated unnecessary descriptive elements within reports.

To gain a more comprehensive picture of standardized reporting initiatives across various countries, it might have been beneficial for the authors to additionally consult with national governing bodies for radiology. These organizations play a central role in disseminating information on standardized reporting templates to radiologists nationwide. While contacting individual radiologists provided valuable insights into their awareness of these practices, it may not fully capture the implementation landscape at a national level. To effectively implement standardized reporting, access to resources often complements radiologist awareness.

However, promising solutions are emerging. Advancements in artificial intelligence have the potential to streamline information dissemination. Additionally, national conferences can play a crucial role in raising awareness among radiologists. Over time, these efforts should ensure these valuable guidelines become more widely adopted within the Indian radiological community.
